# Examination of Aeroallergen Sensitization Patterns in Southeastern Louisiana

**DOI:** 10.31486/toj.25.0049

**Published:** 2025

**Authors:** Margaret Huntwork, Andrew McKernan, Estelle Levetin, Derek Werthmann, Felicia Rabito, William Edward Davis, John C. Carlson

**Affiliations:** ^1^ Section of Clinical Immunology, Allergy & Rheumatology, Tulane University School of Medicine, New Orleans, LA; ^2^ Department of Biological Sciences, University of Tulsa, Tulsa, OK; ^3^ Department of Epidemiology, Tulane University School of Public Health, New Orleans, LA; ^4^ Department of Allergy and Immunology, Ochsner Clinic Foundation, New Orleans, LA; ^5^ The University of Queensland Medical School, Ochsner Clinical School, New Orleans, LA

**Keywords:** Acremonium, *allergens*, *antigens–fungal*, *antigens–plant*, *asthma*, *environmental exposure*, *fungi*, *hypersensitivity*, *immunoglobulin E*, *plant weeds*, *pollen*, *rhinitis–allergic*, *trees*

## Abstract

**Background:**

Episodic assessment of sensitization patterns within a geographic area helps to monitor the role that specific aeroallergens may play in triggering allergic disease. An assessment of sensitization patterns in New Orleans, Louisiana, after Hurricane Katrina identified patterns of sensitization that were different from pre-Katrina studies. Whether the patterns had changed since the post-Katrina assessment was unknown. We therefore sought to evaluate the current sensitization patterns in the greater New Orleans area.

**Methods:**

Seven hundred seventy-six unique patients with at least 1 sensitization identified on a standard 58-allergen skin test panel that includes 24 fungal extracts were evaluated for prevalence, unique sensitization within an allergen group, and patterns of sensitization across patients.

**Results:**

Expected results included a high prevalence of sensitization to house dust mites, grass pollen, and spores of *Alternaria* and *Aspergillus*. Surprising results included a high prevalence of *Acremonium* sensitization and very low sensitization to other fungi. Sensitization patterns for patients did not cluster within phylogenetic patterns for most of the pollen and fungal extracts.

**Conclusion:**

Ongoing refinement of extracts used in skin prick testing will help to monitor changing patterns of sensitization. This monitoring is especially important as climate change, changes in plant cultivation, and urbanization alter ecosystems.

## INTRODUCTION

Allergic rhinitis and allergic asthma are 2 of the most common chronic medical conditions.^1,2^ Worldwide, up to 40% of the population is estimated to be sensitized to at least 1 environmental allergen,^3^ and a 2005 study by Gruchalla et al reported that nearly 95% of the 1,059 children with asthma who were skin tested were sensitized to at least 1 indoor allergen.^4^ Particular allergens have a disproportionate effect on human health.^5-7^ For example, an analysis of the National Health and Nutrition Examination Survey data from 2005 and 2006 found that children with asthma and increasing specific immunoglobulin E (IgE) titers to cockroach, rat, and *Aspergillus* spores had increased emergency department visits, while emergency department visits for adults with asthma were associated with increasing specific IgE levels to 2 species of house dust mites.^6^

Aeroallergen skin prick testing—a percutaneous application of extracts derived from pollen, fungi, insects, rodents, and pets—identifies the allergens to which individuals are sensitized. Skin prick testing for environmental aeroallergens is indicated for patients with chronic rhinitis and persistent asthma.^8^ Information on sensitization can be used on an individual level to identify allergies, guide allergen avoidance and reduction measures, and better categorize a patient's health risks. Unlike indoor allergens, the types and prevalence of outdoor fungal spores and pollen vary geographically, resulting in heterogeneity of skin test panels by region.^9-11^ Aeroallergen skin test panels should be continuously refined based on aerobiologic surveys, allergen prevalence, demographic changes, and available standardized reagents.^8,12^ Because urbanization, weather changes, and changes in plant cultivation may change exposures for people in any given area, local sensitization patterns warrant periodic reevaluation.^9,11^

To determine if changes in our skin test panel were warranted and to reassess drivers of allergic disease in our area, we analyzed aeroallergen sensitization patterns for patients with chronic rhinitis and asthma who underwent allergy testing within our health system. The prior analysis had been conducted more than a decade previously. For comparative purposes, data from the prior study are provided in the Appendix.

## METHODS

After institutional review board approval, we conducted a retrospective review of aeroallergen skin prick testing results from a single, large, regional health system in New Orleans, Louisiana. We included patients who had positive allergy skin prick testing results from tests performed from December 2010 through April 2021. From the electronic medical record, we extracted data on which allergens had positive tests, whether the patient was pediatric (<18 years) or adult (≥18 years) at the time of testing, and whether the patient had a diagnosis of asthma (code J45.XX).

We excluded patients whose skin prick testing was entirely negative and patients whose tests were invalid (ie, positive or negative controls were not positive or negative, respectively). Patients who underwent testing with our complete aeroallergen skin test panel were included, even if their results included missing data. Patients for whom partial panels were conducted include children undergoing testing with multiple devices, typically used in physically smaller children. Patients with partial panels were not included in this analysis.

Skin prick testing was performed using Duotip-Test II devices (Lincoln Diagnostics, Inc) by allergy clinic nurses, allergy fellows, or allergists; all results were read and documented by allergists or allergy fellows. We defined a positive test as a ≥3-mm wheal compared to the negative control; the negative control is a skin prick test with saline, a substance not expected to produce a wheal. Wheals <3 mm were coded as a negative result. During the study period, the standard panel of aeroallergens consisted of 58 tests, including 5 indoor aeroallergens, 17 trees, 8 weeds, 4 grasses, and 24 fungal species. Skin prick testing extracts came from Stallergenes Greer International AG. Histamine (positive) and saline (negative) controls were placed as part of the routine aeroallergen skin test panel.

We identified the frequency of positive results for each allergen, the frequency with which specific allergens were the only sensitization within the particular allergen group (indoor allergens, trees, weeds, grasses, and fungal species), and the frequency of multisensitization. Pearson correlation coefficients were generated using SPSS Statistics version 19.0 (IBM Corporation) to describe the relationship between test positivity for all possible pairings of extracts.

To look for drivers of cross-sensitization, we performed a hierarchical cluster analysis, identifying concordance among allergens across patients. Cross-sensitization (ie, allergy to multiple species from a single sensitization event) is common because IgE binds to similar proteins present in closely related organisms (called panallergens), resulting in clusters of sensitization that usually match the phylogenetic relationships of the allergen source.^13^ Cluster analysis was performed by using a hierarchical clustering algorithm at each generation of clusters using complete linkage. We performed cluster analysis separately for patients <18 years, patients ≥18 years, and individuals with and without asthma and produced plots of fit statistics and dendrograms. Cluster analyses were conducted using SAS version 9.2 (SAS Institute Inc).

## RESULTS

### Prevalence

We identified the positivity rates of specific aeroallergens to differentiate those that are common from those that are rare. We determined rates of monosensitization for each of the allergens to identify the most important representative allergens within each allergen group (Table).

**Table.** Sensitization to Aeroallergens Among 776 Patients With at Least 1 Positive Result on Skin Prick Testing in New Orleans, Louisiana, December 2010 to April 2021

Allergen Group/Specific Allergen/Number of Tests	Number of Positive Tests (%)	Rate of Monosensitization Within Allergen Group, %	Person Correlation Coefficient With Asthma	Person Correlation Coefficient With Age <18 Years
**Indoor allergens**				
*Dermatophagoides pteronyssinus*, n=773	478 (61.8)	19	0.078^a^	0.067
*Dermatophagoides farinae*, n=774	474 (61.2)	20	0.083^a^	0.063
Cat, n=775	286 (36.9)	42	0.128^b^	0.008
Dog, n=776	155 (20.0)	9	0.167^a^	0.059
Cockroach, n=776	174 (22.4)	25	0.009	–0.012
**Trees**				
Pecan, n=775	238 (30.7)	11	0.070^a^	0.109^b^
Birch, n=775	219 (28.3)	0	0.058	0.136^b^
Cypress, n=775	219 (28.3)	0	0.058	0.136^b^
Cedar, n=775	219 (28.3)	11	0.051	0.106^b^
Willow, n=776	196 (25.3)	12	0.043	0.118^b^
Box elder, n=775	194 (25.0)	7	0.046	0.114^b^
Sycamore, n=775	174 (22.5)	3	0.050	0.129^b^
Mulberry, n=772	169 (21.9)	8	0.066	0.117^b^
Elm, n=772	168 (21.8)	3	0.094^b^	0.118^b^
Ash, n=774	163 (21.1)	7	0.050	0.116^b^
Pine, n=776	160 (20.6)	1	0.038	0.111^b^
Hackberry, n=775	153 (19.7)	8	0.066	0.072^a^
Walnut, n=775	147 (19.0)	3	0.075^a^	0.100^b^
Oak, n=776	143 (18.4)	9	0.109^b^	0.086^a^
Cottonwood, n=775	142 (18.3)	6	0.075^a^	0.049
Maple, n=776	140 (18.0)	11	0.025	0.101^b^
Sweetgum, n=776	52 (6.7)	1	0.005	–0.006
**Weeds**				
Mugwort, n=776	222 (28.6)	0	0.016	0.051
Ragweed, n=776	222 (28.6)	0	0.016	0.051
Sorrell/dock, n=775	198 (25.6)	21	0.008	0.106^b^
Pigweed, n=776	184 (23.7)	16	0.028	0.078^a^
Lambs quarters, n=776	166 (21.4)	20	0.046	0.131^b^
Russian thistle, n=776	155 (20.0)	13	–0.015	0.069
Marsh elder, n=776	154 (19.9)	10	0.047	0.041
English plantain, n=775	151 (19.5)	5	0.051	0.114^b^
**Grasses**				
Timothy, n=775	310 (40.0)	37	0.077^a^	0.145^b^
Johnson, n=775	282 (36.4)	17	0.090^a^	0.134^b^
Bermuda, n=776	196 (25.3)	17	0.019	0.127^b^
Bahia, n=776	175 (22.6)	18	0.032	0.052
**Fungal spores**				
*Alternaria*, n=775	347 (44.8)	33	0.110^b^	0.107^b^
*Acremonium*, n=775	282 (36.4)	6	0.090^a^	0.134^b^
*Aspergillus*, n=773	278 (36.0)	12	0.080^a^	0.163^b^
*Cladosporium*, n=776	129 (16.6)	14	0.070	0.034
*Bipolaris*, n=776	114 (14.7)	9	0.157^b^	0.178^b^
*Mucor*, n=775	85 (11.0)	4	0.109^b^	0.108^b^
*Candida*, n=776	67 (8.6)	6	0.072^a^	0.025
*Gliocladium*, n=776	62 (8.0)	2	0.034	0.093^b^
*Rhizopus*, n=775	57 (7.4)	3	0.033	0.062
*Chaetomium*, n=776	54 (7.0)	1	0.041	0.006
*Neurospora*, n=776	54 (7.0)	1	0.055	0.068
*Fusarium*, n=776	54 (7.0)	0	0.068	0.114^b^
*Rhodotorula*, n=776	51 (6.6)	1	0.049	0.060
*Pullularia*, n=775	50 (6.5)	1	0.066	0.094^b^
*Penicillium*, n=776	50 (6.4)	0	0.010	0.062
Smuts, n=776	49 (6.3)	1	0.055	0.097^b^
*Helminthosporium*, n=734	44 (6.0)	3	0.014	0.086^a^
*Epicoccum*, n=775	36 (4.7)	2	–0.032	–0.026
*Botrytis*, n=776	35 (4.5)	0	0.003	0.014
*Curvularia*, n=776	34 (4.4)	0	0.006	0.016
*Phoma betae*, n=775	32 (4.1)	2	0.012	0.041
*Trichoderma*, n=776	24 (3.1)	2	–0.020	0.024
*Stemphylium*, n=770	22 (2.9)	0	0.071^a^	0.007
*Trichophyton*, n=775	17 (2.2)	0	0.028	0.051

^a^*P*<0.05

^b^*P*<0.01

Note: Absolute correlation coefficient values <0.3, including those in this analysis, are considered weak or poor correlations.

#### Indoor Allergens.

Sensitivity to house dust mites (*Dermatophagoides pteronyssinus and D farinae)* occurred most frequently, with 61.5% percent of patients having positive skin prick testing. The 2 species were tested separately, and the prevalence of sensitization was similar. Cat sensitization had 36.9% prevalence, dog was 20%, and cockroach 22.4%. Sensitization to house dust mite, cat, and dog had statistically significant but small correlations with asthma. Cat had the highest monosensitization at 42%; monosensitization to house dust mites occurred in 20% of patients.

#### Trees.

Pecan, birch, cypress, cedar, and willow were the most frequently positive tree pollen. Pecan, elm, walnut, oak, and cottonwood correlated with asthma. Nearly all of the trees correlated with age <18 years. Among patients sensitized to only 1 type of tree pollen, monosensitization occurred in 11% to 12% of patients for pecan, cedar, willow, and maple tree pollen.

#### Weeds.

Mugwort, ragweed, and sorrel/dock were the most frequently positive weed pollens. No weed pollen correlated with asthma. Sorrell/dock, pigweed, lambs quarters, and English plantain correlated with age <18 years. Among patients sensitized to only 1 type of weed pollen, monosensitization occurred most frequently with sorrel/dock and lambs quarters at 21% and 20%, respectively.

#### Grasses.

Timothy grass sensitization had a 40% prevalence, highest of all the pollen tested. Timothy grass and Johnson grass sensitization correlated with asthma, and Timothy grass, Johnson grass, and Bermuda grass sensitization correlated with age <18 years. Among patients sensitized to only 1 of the 4 grass pollen types, monosensitization to Timothy grass occurred in 37%.

#### Fungal Spores.

Among the 725 tested patients with complete data, 463 of 725 patients (63.9%) were sensitized to at least 1 fungal extract. *Alternaria* had the highest fungal spore sensitization prevalence at 44.8%. *Acremonium* tied with *Aspergillus* for second most prevalent fungal spore at 36.4 and 36.0%, respectively. *Penicillium* had a low sensitization rate of 6.4%. Sensitization to *Alternaria, Acremonium, Aspergillus, Bipolaris, Mucor, Candida,* and *Stemphylium* correlated with asthma. Sensitization to *Alternaria, Acremonium, Aspergillus, Bipolaris, Mucor, Gliocladium, Fusarium, Pullularia*, smuts, and *Helminthosporium* correlated with age <18 years. Among patients sensitized to only 1 type of fungus, monosensitization occurred most frequently to *Alternaria* (33%).

### Cluster Analysis

**Figure 1.**
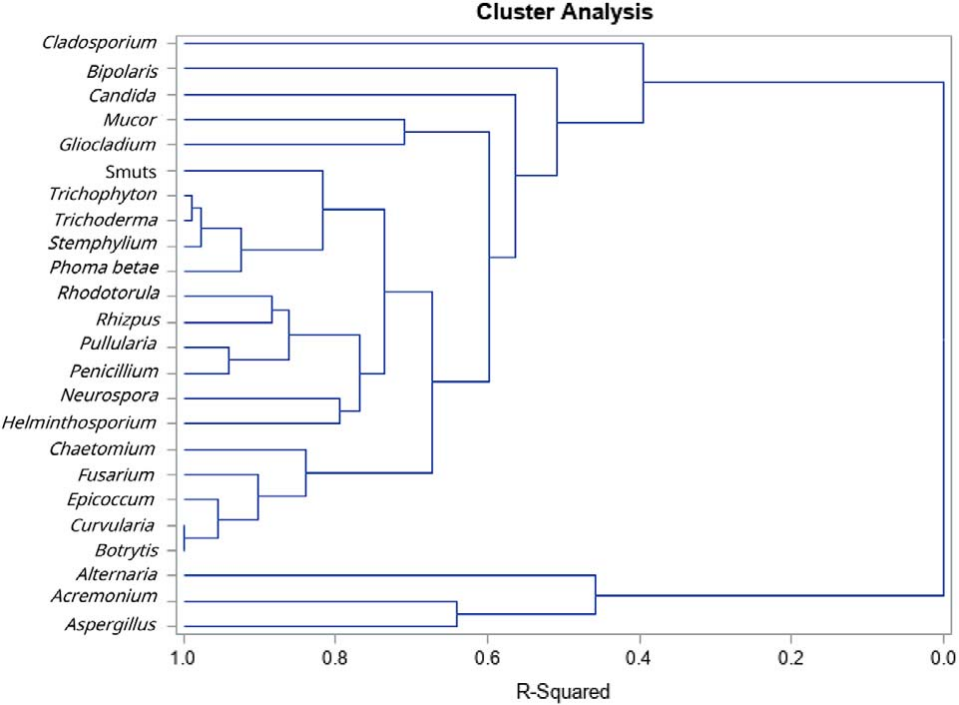
Hierarchical cluster analysis of fungal allergen sensitization did not result in phylogenetically similar clusters.

**Figure 2.**
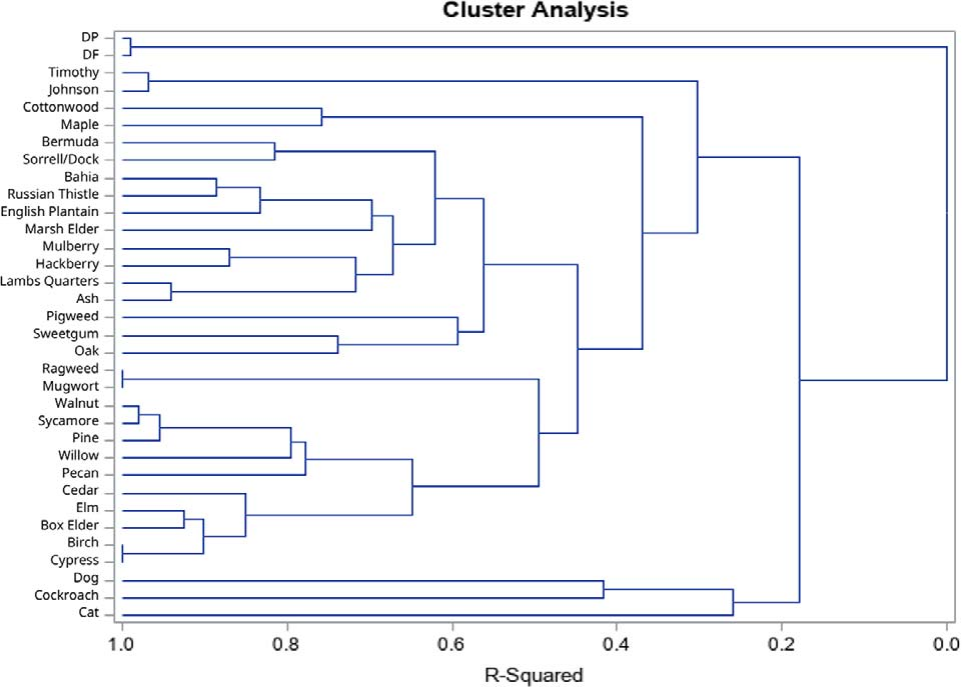
**Hierarchical cluster analysis of allergen sensitization other than fungi did not result in phylogenetically similar clusters for most of the aeroallergens tested.** DF, *Dermatophagoides farinae*; DP, *Dermatophagoides pteronyssinus*

Hierarchical cluster analysis did not result in phylogenetically similar clusters. This pattern persisted when fungi (Figure 1) were separated from other aeroallergens (Figure 2), when data from patients <18 years were separated from data from patients ≥18 years, and when only patients with a diagnosis of asthma were modeled (data not shown).

## DISCUSSION

These results show important changes in aeroallergen sensitization patterns previously identified in southeastern Louisiana. Sensitization to ragweed has varied over the years: the rate of sensitization was 14.5% pre-Katrina,^14^ 17.8% in 2008,^14^ 36.8% in a 2006-2011 assessment by Anthony et al (Appendix), and 28.6% in the current study. While overall sensitization to ragweed has varied, monosensitization to ragweed has decreased: 30% were monosensitized in the Anthony et al 2006-2011 study period (Appendix), and no patients showed monosensitization to ragweed in the current study.

Sensitization patterns vary by type of pollen and timing of exposure. For example, a 2024 Canadian study identified early-life exposure to tree canopy as protective for tree pollen sensitization, while exposure to grass and weed pollen increased risks of sensitization.^15^ Climate change has altered human exposure to these allergens in ways that make predicting outcomes difficult for individual locations.^16^ Thus, ongoing monitoring of pollen exposure and sensitization in different locations is warranted.

Cross-sensitization to allergens from phylogenetically related groups is common.^13^ However, the clustering of sensitizations in our data did not follow phylogenetic relationships as often as expected. The 2 house dust mite species clustered together as expected. However, only 2 of the grasses (Timothy grass and Johnson grass) clustered together, while Bermuda grass clustered with sorrel/dock, and bahia grass clustered with Russian thistle. Ragweed and mugwort clustered together as expected given their mutual placement in the Asteraceae. Birch clustered with the unrelated cypress rather than with oak. This incomplete clustering cannot be easily explained.

More substantial differences were seen in mold sensitization. The aeroallergen skin test panel included fungi normally encountered both indoors and outdoors. Some, like the *Penicillium* and *Aspergillus* species, are known to proliferate in indoor environments and occur at higher concentrations in air samples from indoor environments vs outdoors, especially in locations with indoor moisture issues. *Cladosporium* and *Alternaria* proliferate indoors as well but normally have higher concentrations outdoors. Results for skin prick testing with fungal extracts showed differences when compared to 2 studies conducted in New Orleans following Hurricane Katrina. The current study showed an overall higher rate of positive reactions to fungal allergens (63.9%) than Rabito et al^17^ reported in 2010 (10.4%) and Anthony et al found in their 2006-2011 assessment (32.8%) (Appendix). By contrast, the Head-off Environmental Asthma in Louisiana (HEAL) study generally found higher rates of positive reactions to fungal allergens, with 72% testing positive to at least 1 fungal extract.^18-20^ Some of these differences may be attributable to the use of different extract sources in the studies. The studies also had important demographic differences. The HEAL study analysis included 182 children (4 to 12 years old) who had moderate to severe asthma,^18^ the Anthony et al 2006-2011 assessment included 125 adults with and without asthma (Appendix), and the Rabito et al study included 529 children and adults with and without asthma.^17^ The highest prevalences of fungal sensitization among atopic patients in the current study were for sensitivity to *Alternaria* (44.8%), *Acremonium* (36.4%), and *Aspergillus* (36.0%). The sensitivity to these fungi was similar to the results in the HEAL study.^18^ While *Alternaria and Aspergillus* are well-studied allergens, in general, less is known about the allergenicity of *Acremonium.*^21^

Cross-reactivity among fungal allergens has been recognized for decades. Studies have shown cross-reactivity between various fungi including *Alternaria alternata, Cladosporium herbarum,* and *Epicoccum nigrum*.^22-25^ Allergenic proteins from many fungi have been identified, characterized, and sequenced.^24^ As more fungal allergens have been sequenced, patterns of relatedness among these proteins have been found. Many of the fungal allergens belong to well-studied protein families such as serine protease, enolase, heat shock proteins, and cytochrome C.^26-30^ These shared proteins may result in cross-reactivity. In fact, extensive cross-reactivity has been demonstrated for fungal enolase and serine protease allergens, suggesting that these allergens may be fungal panallergens.^27^

No allergens from *Acremonium strictum* have been characterized or sequenced. In a study of sensitization to indoor fungi in West Virginia, Beezhold et al found that 21 of 102 patients tested positive to at least 1 fungal extract, and 6 patients had a positive skin prick test to *Acremonium*.^21^ The authors performed immunoblotting with patient sera sensitized to *Acremonium* and found multiple proteins that were IgE reactive in *Acremonium*. Possible cross-reactivity with *Alternaria alternata* and *Aspergillus fumigatus* extracts was also examined with immunoblot inhibition. IgE binding to *Acremonium* extract was partially inhibited by both *Alternaria alternata* and *Aspergillus fumigatus* extracts.^21^ Cross-reactivity may partially explain the high percentage of positive reactions seen in the present study.

Research has shown that the genus *Acremonium* is polyphyletic, and species formerly classified in that genus have been reclassified into different genera, families, and orders. The species that is typically used in immunotherapy, *Acremonium strictum,* has been transferred to the genus *Sarocladium* and is now known as *Sarocladium strictum.*^31^ For convenience and clarity, the genus name *Acremonium* is used consistently in this manuscript. *Acremonium* is frequently found in the soil or on decaying vegetation and is introduced indoors where it may proliferate, particularly in wet substrates. *Acremonium* species typically produce small, single-celled, usually colorless conidia in a slimy mass.^32-34^ Lacking distinctive morphologic features, the spores are not usually recognized in spore trap samples but can be detected in culture-based samples. However, like other fungi produced in slime, *Acremonium* conidia tend to stick together and are not readily aerosolized. Nevertheless, in a study of 1,717 buildings across the United States, using culture-based air sampling, Shelton et al recovered *Acremonium* from 137 buildings.^35^ *Acremonium* has been frequently recovered from moldy building materials and indoor dust samples, especially in buildings with moisture damage.^36-40^ Of note is a study by Vesper et al of indoor fungi using a quantitative polymerase chain reaction assay; the authors reported *Acremonium* occurred in vacuum dust samples in 57% of 1,096 US homes.^36^ No data are available on the occurrence of *Acremonium* or other fungi in the homes of patients in the current study.

The prevalence of sensitization to *Penicillium* in the current study is 6.4%. This result/percentage is identical to the rate of sensitization Anthony et al found more than 10 years ago (Appendix) and slightly higher than the 2010 Rabito et al study that reported 1.7% of New Orleans patients with *Penicillium* sensitization.^17^ These rates are notably lower than the sensitization rate reported in the HEAL study, which showed that 48% of patients with asthma aged 4 to 12 years were sensitized to *Penicillium*,^18^ and lower than the sensitization rate found in the Inner City Asthma Study, which showed that 13% of pediatric patients with asthma were sensitized to *Penicillium*.^41^ In our study, *Penicillium* sensitization was not correlated with the group of patients <18 years or with patients diagnosed with asthma. Additionally, we did not find any patients monosensitized to *Penicillium*.

Our clinic uses an expanded skin test panel of mold extracts because of concern that mold exposure has increased with the frequent flooding in our area of the country. The data in this study provide reassurance that sensitization to most of these fungi is rare. Testing may be considered for rare sensitization when clinical presentations suggest that identifying these sensitizations is important.^42^

Study limitations include the exclusion of patients receiving partial panels (particularly reducing the number of younger children included) and the inability to include patients who received allergy testing via serum assays.

Episodic assessment of aeroallergen sensitization patterns is useful for maximizing the value of allergenic extracts used. Reassessment will be especially important as climate change increases global temperatures and extreme weather events. While the Rabito et al study did not identify changes in fungal sensitization after the 2005 flooding of Hurricane Katrina,^17^ ongoing changes may ultimately lead to differences. Assessment of sensitization patterns would ideally be coupled with ongoing evaluation of exposures in the form of air sampling for pollen and spores in outdoor air, as well as indoor sampling for exposure to species of dust mites, such as *Blomia tropicalis*, and cockroaches, including the *Periplaneta* species.

## CONCLUSION

Aeroallergen sensitization patterns for patients undergoing skin prick testing in southeastern Louisiana had high rates of sensitization to *Acremonium* in addition to more expected allergens. Patterns of sensitization did not follow the patterns of cross-sensitization expected with panallergens and differed in important ways from an assessment performed in 2010. Ongoing periodic assessment is warranted to ensure testing is performed for the most relevant triggers of asthma and allergic rhinitis.

## Figures and Tables

**Appendix Figure.**
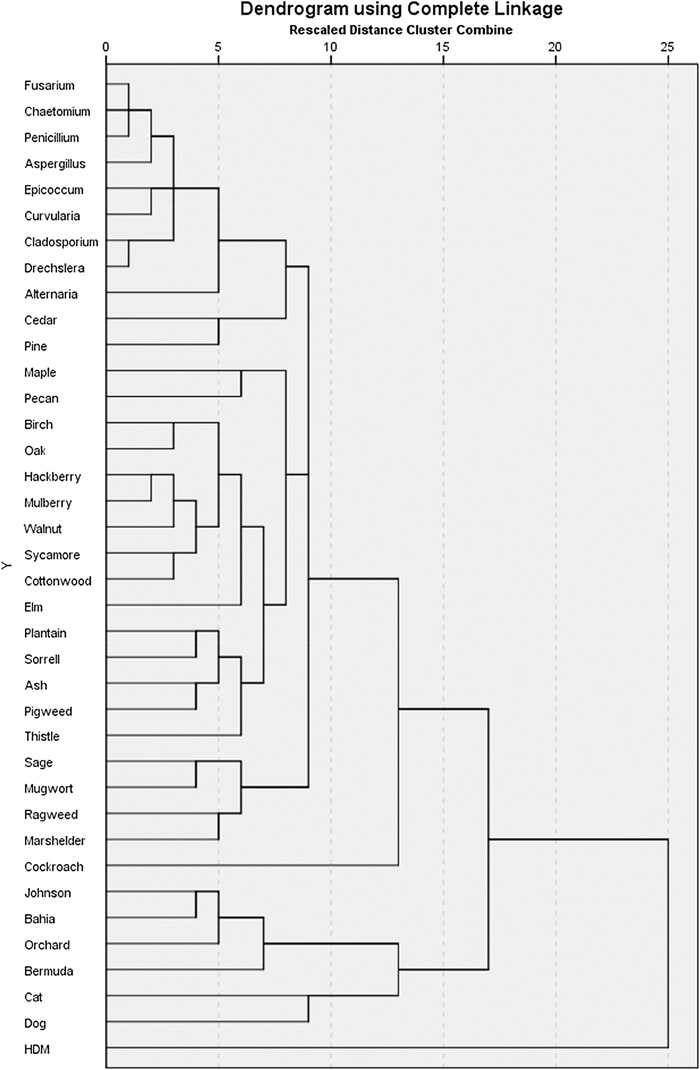
**Dendrogram made from a hierarchical cluster analysis of allergen sensitization using furthest neighbor clustering method.** HDM, house dust mite.

## Notes

*This article meets the Accreditation Council for Graduate Medical Education and the American Board of Medical Specialties Maintenance of Certification competencies for Patient Care, Medical Knowledge, and Practice-Based Learning and Improvement.*

## Appendix. Aeroallergen Sensitization Patterns in New Orleans, 2006 to 2011

These data were presented at the 2012 annual meeting of the American College of Allergy, Asthma & Immunology in Anaheim, California, and published in Anthony K, Carlson JC, Wild LG. Identifying aeroallergen sensitization patterns in New Orleans for improved aeroallergen extract selection. *Ann Allergy Asthma Immunol.* 2012;109(5):A26.

For this project, after institutional review board approval, we examined data from 125 patients with complete aeroallergen skin prick tests and at least 1 positive result performed from 2006 to 2011 in an allergy clinic located in southeastern Louisiana. Skin prick tests used 4 indoor allergens, 13 tree, 8 weed, 4 grass, and 9 fungal extracts manufactured by ALK-Abelló A/S. We determined the frequency of sensitization to allergenic extracts and calculated the rate of monosensitization within the trees, weeds, grasses, and fungi groups.

Hierarchical cluster analysis, using SPSS Statistics version 19.0 (IBM Corporation), grouped sensitization to allergens into a dendrogram, using furthest neighbor clustering method (selected due to the effects of outliers from phylogenetically distant allergen sources).

Results are shown in the Appendix Table and the Appendix Figure.

**Appendix Table.** Sensitization to Aeroallergens Among 125 Patients With at Least 1 Positive Result on Skin Prick Testing in New Orleans, Louisiana, 2006 to 2011

Allergen Group/Specific Allergen	Positive Tests, %	Rate of Monosensitization Within Allergen Group, %
**Indoor allergens**		
House dust mite mix	78.4	N/A
Cat	58.4	N/A
Dog	32.0	N/A
Cockroach	32.0	N/A
**Tree pollen (n=94 patients with any positive skin prick test to tree pollen; 30 patients with only 1 tree sensitization)**		
Pecan	36.8	6.7
Maple	34.4	23.0
Oak	32.8	6.7
Birch	32.8	6.7
Cottonwood	27.2	3.3
Ash	27.2	10.0
Sycamore	26.4	3.3
Elm	25.6	13.0
Walnut	23.2	3.3
Cedar	20.0	23.0
Mulberry	20.0	0.0
Hackberry	20.0	0.0
Pine	10.4	0.0
**Weed pollen (n=82 patients with any positive skin prick test to weed pollen; 23 patients with only 1 weed sensitization)**		
Ragweed	36.8	30.0
Marsh elder	33.6	17.4
Pigweed	30.4	8.7
English plantain	30.4	4.0
Sage	29.6	8.7
Sorrell/dock	27.2	13.0
Russian thistle	27.2	13.0
Mugwort	27.2	4.0
**Grass pollen (n=91 patients with any positive skin prick test to grass pollen; 29 patients with only 1 grass sensitization)**		
Orchard	51.2	34.0
Bermuda	49.6	41.0
Bahia	49.6	17.2
Johnson	40.8	6.9
**Fungal spores (n=41 patients with any positive skin prick test to fungal spores; 15 patients with only 1 fungal sensitization)**		
*Alternaria*	20.0	66.0
*Epicoccum*	16.0	6.7
*Drechsleria*	13.6	6.7
*Cladosporium*	11.2	6.7
*Aspergillus*	9.6	6.7
*Curvularia*	8.0	6.7
*Penicillium*	6.4	0.0
*Fusarium*	4.8	0.0
*Chaetomium*	3.2	0.0

N/A, not applicable.
